# Response to the Letter regarding “The usefulness of the genetic panel in the classification and refinement of diagnostic accuracy of Mexican patients with Marfan syndrome and other connective tissue disorders”

**DOI:** 10.17305/bb.2024.10836

**Published:** 2024-10-01

**Authors:** Ricardo Gamboa, Maria Elena Soto

**Affiliations:** 1 Department of Physiology, Instituto Nacional de Cardiología Ignacio Chávez, Mexico City, Mexico; 2 Research Direction, Instituto Nacional de Cardiología Ignacio Chávez, Mexico City, Mexico

Dear Editor,

In relation to the letter expressing concerns about some important points of the article entitled “The usefulness of the genetic panel in the classification and refinement of diagnostic accuracy of Mexican patients with Marfan syndrome and other connective tissue disorders” [1], we would like to comment on the following.

Regarding the importance of the participation of a clinical geneticist, we understand physicians’ concerns about collaborating with a clinical geneticist. The clinical assessment and interpretation of the patients’ conditions studied was carried out at the National Institute of Cardiology in Mexico by an interdisciplinary group, including cardiologists, ophthalmologists, orthopedists, rheumatologists, geneticists, and psychologists. These specialists collectively classified patients according to clinical and genetic criteria. The head of this working group is Dr. Maria Elena Soto, a rheumatologist specialized in this type of connective tissue disease and its relationship with cardiovascular diseases, particularly the aorta. Dr. Soto has over 20 years of experience in this field and we have collaborated with her on numerous high-level scientific publications on the subject [2–5]. This assures us of the accuracy of our patient classification, as mentioned in our methodology. On the other hand, this project was approved by the ethics committee, ensuring our patients’ correct classification.

Although a specialist in clinical genetics is required, as the doctors mentioned, the proper classification of our patients was achieved through interdisciplinary work. It is important to note that our work is focused on cardiovascular diseases, particularly on problems of the aorta, which is one of the main clinical concerns due to its lethality. Therefore, we did not focus on hypermobile spectrum disorders. Additionally, it is essential to mention that our group also included specialists in molecular biology who carried out the sequencing, analysis, and interpretation of results.

A second concern mentioned regards the phenotype-genotype correlation and the use of the 174-gene panel, considering there are currently 545 genes associated with joint hypermobility. We agree that there are additional genetic associations and that reaching a definitive conclusion is challenging. While more than 545 related genes exist, as the doctors mention, we specifically analyzed genes related to cardiovascular problems using TruSight Sequencing panels–Illumina-cardiovascular. Our objective was to investigate the phenotype–genotype relationship in aortic damage, focusing on the most relevant genes, as shown in Figure 1. Our study did not focus on hypermobile spectrum disorders (HSD). We understand the importance of sequencing and studying all genes: however, the associated costs are high in our situation.

As mentioned in the conclusion of our work, the correlation between phenotype and genotype in these disorders, as assessed by the genetic panel, is complex due to significant heterogeneity in both phenotype–genotype associations and the diversity of aortic and cardiovascular damage. A future perspective could involve expanding the multipanel to include genes related to musculoskeletal, ocular, and metabolic impairments in these syndromes.

A third question concerns the phenotype–genotype correlation and the criteria used in classifying disease severity as mentioned in [6]. This is explained in the methods section. Different programs were taken into account and used (Figures 3 and 4, Table 4): “Using NGS, both germline and somatic variants can be characterized for individual patients. After obtaining the aligned and assembled sequences, variant calling was performed using DRAGEN Enrichment software. Variants were annotated using Illumina’s ‘Variant Interpreter’ server (https://variantinterpreter.informatics.illumina.com/home). Only variants that passed the quality control (QC) metrics and had a frequency greater than 0.01 in the TOPmed, 1000 Genomes Project, and NHLBI Exome Sequencing Project were considered. Utilizing the ClinVar database (https://www.ncbi.nlm.nih.gov/clinvar/), which aggregates genomic variation and its relation to human health, we classified the identified genetic variants as pathogenic, likely pathogenic, or variants of unknown significance (VUS). These classifications follow the standards and guidelines for the interpretation of sequence variants as recommended by the consensus of the American College of Medical Genetics and Genomics and the Association for Molecular Pathology [6].” This is the same reference mentioned in the letter.

The last question concerned the study’s approach not mentioning hypermobile spectrum disorders and Ehlers–Danlos syndromes (EDS). Ignoring these diagnoses can lead to underestimations in prevalence and incomplete therapeutic approaches. However, as mentioned in the article and according to our study population, we analyzed ten patients with Ehlers–Danlos (Table 1); due to their cardiovascular problems. Our objective, as stated earlier, was not focused on hypermobile disorders.

Finally, this work showed that genetic testing provides greater diagnostic certainty than relying solely on clinical aspects. Additionally, identifying the severity and type of genetic variant can inform clinical decision making for patients who may require interventional or surgical cardiovascular treatment.

We appreciate your observations, which are always valuable and important, and we hope to have addressed your questions adequately.
